# Comment on “Differential Effects of MitoVitE, α-Tocopherol and Trolox on Oxidative Stress, Mitochondrial Function and Inflammatory Signalling Pathways in Endothelial Cells Cultured under Conditions Mimicking Sepsis. *Antioxidants* 2020, *9*(3), 195”

**DOI:** 10.3390/antiox9060462

**Published:** 2020-05-29

**Authors:** Karl J. Hegarty, Frances L. Byrne

**Affiliations:** 1 School of Biochemistry and Immunology, Trinity Biomedical Sciences Institute, Trinity College Dublin, D02 Dublin, Ireland; hegartka@tcd.ie; 2 School of Biotechnology and Biomolecular Sciences, University of New South Wales, Sydney, NSW 2052, Australia

Minter, B.E. et al. recently published an article titled “Differential Effects of MitoVitE, α-Tocopherol and Trolox on Oxidative Stress, Mitochondrial Function and Inflammatory Signalling Pathways in Endothelial Cells Cultured under Conditions Mimicking Sepsis” [1]. The authors used a model of sepsis that involves treating endothelial cells with bacterial glycopolymers to trigger oxidative stress and inflammation. Using this model, they investigated three different Vitamin E compounds that differ in structure, function, and cellular localisation.

The authors found that all compounds had antioxidant effects, as expected; however, the mitochondria-targeted form of vitamin E (mito-VitE) had by far the most profound anti-inflammatory profile. Despite the clearly superior beneficial effect of mito-VitE to lower expression of proinflammatory genes, the authors make the confusing interpretation that their results “challenge the concept that protection inside mitochondria provides better protection” in conditions mimicking sepsis.

This study has several limitations that should also be noted:

1. The effects of vitamin E compounds on Alamar Blue (resazurin) metabolism should not be used as the only indication of “mitochondrial function”, because Alamar Blue has a redox-based mechanism. At a minimum, a direct readout of mitochondrial oxidative metabolism, ATP-dependent respiration, and spare respiratory capacity in a mitochondrial stress test would be needed to make conclusions about mitochondrial function.
2. The focus of this article was to determine the effects of “compartmentalised antioxidants” on different parameters, including oxidative stress. The authors used carboxy-DCFDA to measure oxidative stress. However, readouts from this dye do not provide information on subcellular location. Other dyes may have been more useful, including a mito-specific dye such as MitoSOX Red. A complimentary approach would be to measure the dimerization/oxidation of ROS-sensitive peroxiredoxins (PRDX) that occur in different subcellular compartments. Specifically, PRDX3 is localised to mitochondria and PRDX2 is found in the nucleus and cytoplasm [2]. These experiments would provide valuable information about how cells respond to LPS/PepG and how each of the antioxidants (MitoVitE, α-Tocopherol and Trolox) alter this phenotype in these subcellular locations.
3. Only one concentration of each vitamin E derivative was tested. It is well known that mito-VitE accumulates by orders of magnitude in mitochondria [3], and thus the localised concentrations could mean that mito-VitE has an efficacy/toxicity ratio that is different from other compounds.
4. There is no control gene expression for each vitamin E form under normal conditions (in the absence of LPS/PepG), so the upregulation and/or downregulation of genes is likely masked by stimulation with LPS/PepG.
5. The authors described α-tocopherol as the “most biologically active” vitamin E form, which is misleading since vitamin E forms γ-tocotrienol, γ-tocopherol, and δ-tocopherol have all demonstrated superior antioxidant and anti-inflammatory properties, and would have been better comparisons for this experiment [4,5]. As well as this, Trolox was described as a “potent antioxidant” by the authors, despite the protective ability of Trolox in oxidative damage previously being compared to mito-VitE (MitoE2, MitoE10), where it was shown to be much less protective [6,7].
6. The data on inflammation is minimal and could be markedly improved with examination of inflammasome activation. The data show that neither α-tocopherol nor Trolox appear to be protective by the same mechanism as mito-VitE, which sustained mitochondrial membrane potential at a level similar to vehicle control cells. α-tocopherol and Trolox significantly increase expression of PTGS2 (COX2), which is responsible for prostanoid biosynthesis (e.g., PGE2), while mito-VitE downregulated the expression of PTGS2; however, the reasons for this are neither discussed nor investigated further. Non-canonical inflammasome (caspase-11) activation has been shown to be more important than Caspase-1 in sepsis [8], and was inhibited by PGE2 in asthma [9]. NLRP3 inflammasome activation is also achieved through caspase-11 induction [10] and mtROS production [11], and has been shown to be inhibited by PGE2 [12].

These connections are missing in the present paper but may be vitally important to consider in the context of concluding whether mitochondrial redox protection is more or less superior to nontargeted approaches.
